# Proficiency of South African intern doctors in recognising ischaemia on an electrocardiogram

**DOI:** 10.4102/jcmsa.v3i1.190

**Published:** 2025-05-30

**Authors:** Relisha K. Naidoo, Duncan M. Havenga, David Morris

**Affiliations:** 1 Division of Emergency Medicine, Faculty of Health Sciences, University of KwaZulu-Natal, Durban, South Africa

**Keywords:** electrocardiogram, ischaemia, interpretation, assessment, competency, accuracy, self-confidence, intern doctors

## Abstract

**Background:**

Interpreting an electrocardiogram (ECG) is challenging for junior doctors, and proficiency gaps exist commonly worldwide. This study assessed interpretation accuracy and self-confidence of South African intern doctors in identifying acute myocardial ischaemia on an ECG and analysed the impact of an education strategy.

**Methods:**

This descriptive, quantitative study included a prospective analytical component using a standardised teaching session. All participants completed an ECG interpretation test and self-confidence questionnaire both before and after the teaching session. Descriptive statistics summarised demographics; data analyses included means, medians, modes, and individualised comparisons of pre- and post-teaching test scores and self-confidence.

**Results:**

One hundred and sixty-four interns were analysed. At baseline, interns demonstrated above-average accuracy (mean test score 60.1%) and most declared moderate insecurity with ECG interpretation. Post-teaching, accuracy improved marginally (mean test score 65.8%) (*p* < 0.001), and self-confidence improved drastically with most becoming moderately confident (*p* < 0.001). However, while there was a significant improvement in the recognition of four ischaemic and two non-ischaemic ECG patterns, the misinterpretation of one non-ischaemic ECG significantly worsened, and there was an insignificant impact on half of the ischaemic ECGs tested.

**Conclusion:**

Interns demonstrated above-average accuracy in identifying acute myocardial ischaemia on an ECG with moderate insecurity at baseline. While a teaching session improved self-confidence, its impact on accuracy was mixed. This highlights a need for ongoing training of South African intern doctors in ECG interpretation.

**Contribution:**

This study was the first to assess ECG interpretation accuracy and associated self-confidence of South African intern doctors, thereby offering a local perspective on this essential skill.

## Introduction

An electrocardiogram (ECG) plays a pivotal role in the initial assessment of a patient presenting to the emergency department with suspected acute coronary syndrome.^1^ Electrocardiogram machines are widely available and commonly used at many healthcare facilities in South Africa, but the diagnostic impact of an ECG hinges greatly on the proficiency and associated self-confidence of its interpreter. The interpretation of an ECG is a complex skill that requires the astute integration of cardiovascular anatomy, physiology and pathology core knowledge with electrophysiological pattern recognition in the patient’s clinical context.^2,3^ Acquiring competency in ECG interpretation is critical for all doctors, especially in the emergency department, as this skill facilitates the timeous and accurate identification of acute myocardial ischaemia, and the distinction of it from other conditions that may mimic its clinical presentation.

The Health Professions’ Council of South Africa (HPCSA) recognises ECG interpretation as an essential skill for newly qualified doctors to practise and consolidate during internship.^4^ Internship training spans a period of 2 years during which interns rotate within multiple specialities at accredited facilities with the objective of acquiring the skills that embody a safe and competent medical practitioner. Therein lies the HPCSA requirement for all interns to complete a minimum of 10 supervised ECG interpretations as part of a training logbook to demonstrate competency.^4^ However, ECG interpretation skills are often deemed a challenge to both teach and acquire.^5,6^ Currently, no standardised programme exists to monitor the quality or consistency of teaching of this skill during internship training across hospitals.

Several studies evaluating ECG interpretation skills among interns in other countries revealed moderate-to-low competency levels,^7,8,9,10^ with some interns attributing these proficiency gaps to inadequate training received at medical school.^8,9^ A study conducted at a Peruvian university found that the majority of their interns demonstrated a medium-to-high skill level, and this was strongly associated with better academic performance at medical school.^11^ In addition, interns commonly expressed low self-confidence in ECG interpretation.^8,9^ In a study that explored the reasons behind the fear of ECG interpretation, most medical students and junior doctors attributed their lack of confidence to not receiving enough teaching and supervised practice during their training years.^12^

There is limited research evaluating ECG interpretation accuracy and self-confidence among doctors practising in South Africa. A study by De Jager et al. in 2010 revealed a low level of accuracy in ECG interpretation among emergency medicine residents and newly qualified emergency physicians across South Africa.^13^ Similarly, a study published in 2020 by Mabuza and Mntla identified poor competency among generalist practitioners of varying seniority across South Africa.^14^ However, neither of these studies evaluated the impact of a standardised ECG training session on these baseline ECG interpretation skills. In addition, despite a reliance on junior doctors to manage emergencies, particularly at rural clinics and district hospitals during night shifts, there are currently no studies assessing ECG interpretation accuracy and associated self-confidence of South African intern doctors specifically. Our study hypothesised that intern doctors in South Africa interpret ECGs with inadequate accuracy and low self-confidence.

The aim of this study was to assess the interpretation accuracy and self-confidence of South African intern doctors at a state hospital-complex in identifying acute myocardial ischaemia on an ECG. In addition, this study evaluated the impact of a standardised teaching session on the baseline accuracy and self-confidence of interns in ECG interpretation, comparing the results before and after the teaching session.

## Research methods and design

### Study design and setting

This was a descriptive, quantitative study that included a prospective analytical component using a standardised teaching session. The study was conducted at the Pietermaritzburg hospital-complex, which serves the uMgungundlovu health district located in the province of KwaZulu-Natal, South Africa. This state hospital-complex is accredited by the HPCSA for internship training and comprises of three hospitals: Greys Hospital (tertiary level), Harry Gwala Regional Hospital (regional level) and Northdale Hospital (district level).

### Study population

This study included the first-year and second-year intern doctors that were rotating within the Pietermaritzburg hospital-complex for their HPCSA-mandated internship training during the data collection period, which was from 28 December 2023 to 23 February 2024. Interns were only allowed to participate if present in-person at a data collection session. Interns were excluded from the study if they were not willing to participate, if their data collection booklets were incomplete or illegible, or if they were unwilling to keep the study’s content concealed from interns who were yet to participate.

### Sampling strategy

A convenience sampling strategy was applied to facilitate data collection using a pragmatic approach. All the department heads (General Medicine, General Surgery, Anaesthesiology, Obstetrics and Gynaecology, Paediatrics, Psychiatry, Orthopaedics and Family Medicine) were approached to schedule data collection sessions with the interns rotating within their respective specialities. Multiple sessions were scheduled within some departments to accommodate for initial non-attendance and help minimise selection bias. Based on an estimated population of 220 interns, an initial target of 192 interns was deduced to detect a significant difference made by the study’s ECG teaching session on the interns’ baseline interpretation accuracy and self-confidence. During data collection, approximately 200 interns were rotating within the Pietermaritzburg hospital-complex, and only 164 interns participated in the study.

### Electrocardiogram teaching session

The ECG teaching session in this study was standardised to ensure that each participant received identical content, instruction and delivery. This standardisation refers specifically to the uniformity of the session across all participants. The session lasted approximately 30 min, with no variation in the material presented or the teaching method used. The teaching session was compiled by the primary investigator, refined by the coauthors and subsequently verified by three external emergency medicine specialists before its implementation. A computer-assisted, lecture-based educational approach was selected for its feasibility, reproducibility and effectiveness in standardising content delivery. Its learning objectives were defined to align with the study’s ECG tests. It took the format of a Microsoft PowerPoint slideshow presentation (Microsoft Corporation. Microsoft PowerPoint [Internet]. 2018. Available from: https://office.microsoft.com/powerpoint). The slideshow was presented to the participants using a classroom projector and included a pre-recorded audio delivered by the primary investigator to complement the visual content. It first reviewed the systematic approach to interpreting any ECG using a didactic style of instruction before turning its focus to the identification of acute myocardial ischaemia by taking the participant through a stepwise approach in recognising red flag patterns and differentiating these from common ischaemia mimics. All participants were exposed to the teaching session during a data collection session, and collective comparisons were made between the participant data measured before and after the teaching session.

### Data collection

All data collection sessions were conducted and invigilated by the principal investigator in-person. Study enrolment necessitated interns to be present in-person and to begin by signing a combined participant informed consent and non-disclosure agreement (Appendix 1). The non-disclosure clause prohibited participants from sharing the content of the study with interns who were yet to participate. Data collection booklets were paper-based, and each booklet comprised of a pre-teaching session questionnaire, a pre-teaching session ECG interpretation test, a post-teaching session questionnaire and a post-teaching session ECG interpretation test. Each data collection booklet contained a unique reference number which then represented an individual participant. An electronic attendance record guarded against duplicate enrolment by using participants’ HPCSA IN (intern) registration numbers. However, participant anonymity was still maintained, as this electronic record was extraneous to the participants’ paper-based data collection booklets.

Participants first completed the pre-teaching session questionnaire followed by the pre-teaching session ECG interpretation test. The questionnaire collected demographical data and explored baseline self-confidence in ECG interpretation using a Likert scale. The ECG interpretation test comprised of 12 computer-generated ECG traces that were selected from a textbook designed for studying ECG interpretation with permission from the authors.^15^ The test combined ECGs depicting true acute myocardial ischaemia with others that reflected a different diagnosis, thereby assessing the interns’ accuracy in identifying acute myocardial ischaemia using ECG interpretation skills. The 12 ECG patterns tested included inferior ST-elevation myocardial infarction (STEMI) with right ventricular involvement, inferior STEMI with posterior extension, lateral STEMI, non-ST-elevation myocardial infarction (NSTEMI) involving anterolateral and inferior territories, Wellens syndrome Type A, Wellens syndrome Type B, hyperacute T-waves, left bundle branch block (LBBB) meeting modified Sgarbossa–Smith criteria, ventricular-paced rhythm not meeting modified Sgarbossa–Smith criteria, normal sinus rhythm, benign early repolarisation (BER) and acute pericarditis. In the test, participants were instructed to identify the ECGs that represented acute myocardial ischaemia by marking an ‘X’ on those ECG paper traces. If participants did not identify ischaemia on a particular ECG, instructions were given to leave that ECG paper trace blank. The pre-teaching session questionnaires and ECG tests were collected from the participants upon completion and sealed in an opaque box by the principal investigator to ensure confidentiality and prohibit alteration of answers. The ECG teaching session then immediately followed, and all participants received it at their respective data collection sessions.

Immediately following the ECG teaching session, participants completed the post-teaching session questionnaire and post-teaching ECG interpretation test all in the same sitting. The post-teaching session questionnaire prompted participants to reassess their self-confidence in ECG interpretation and to rate the helpfulness of the study’s ECG teaching session using Likert scales; in addition, participants were asked if they felt they needed more formal teaching around ECG topics in general during their internship training. The post-teaching session ECG test comprised of the same 12 ECGs used in the pre-teaching session test but compiled in a shuffled order. Upon completion, the post-teaching session questionnaires and ECG tests were sealed in a second opaque box by the principal investigator, and this concluded the data collection session. The reference numbers unique to each data collection booklet were then used to reunite each participant’s pre-teaching session and post-teaching session tests and questionnaires thereby facilitating individualised comparisons and data analyses. Although no separate control group was included, each participant effectively acted as their own control. Pre-teaching test scores served as a baseline comparator against post-teaching test scores, allowing for individualised assessment of improvement following the teaching session.

The decision to conduct the pre-teaching and post-teaching data collection sessions in the same sitting was made for practical and methodological reasons. Given the in-person nature of data collection, this approach ensured that all participants completed both assessments, thereby reducing the risk of participant dropout and minimising attrition bias. Conducting sessions in-person also added validity to the response process, ensuring that answers were self-reported without external influence.

### Data analysis

Electrocardiogram interpretation tests were marked by the primary investigator using the textbook’s model answer, with each participant obtaining a pre-teaching session and post-teaching session test score.^15^ Each test score reflected the number of ECGs that were correctly identified as acute myocardial ischaemia, and no negative marking was applied. All test scores and questionnaire data were manually captured by the primary investigator onto an electronic Microsoft Excel document that was password-protected (Microsoft Corporation. Microsoft Excel [Internet]. 2018. Available from: https://office.microsoft.com/excel). Data entries were cross-checked by the primary investigator using a single-person double-entry approach.

The data were analysed using IBM SPSS Statistics (Version 29) predictive analytics software, and a *p* value of less than or equal to 0.05 was considered significant. Descriptive statistics were employed to summarise participant demographic characteristics, including frequencies and percentages for categorical data. The frequency distribution of numerical data was analysed for means, medians and modes. The same interns were assessed before and after the ECG teaching session, and individualised comparisons were made using the Wilcoxon signed-rank test to analyse the impact of the teaching session on the interns’ baseline ECG interpretation test scores and self-confidence levels.

### Ethical considerations

Ethical clearance to conduct this study was obtained from the University of KwaZulu-Natal Biomedical Research Ethics Committee (No. BREC/00005193/2023), the National Department of Health of South Africa (NHRD Ref:KZ_202308_011), and the management and ethics committees of Greys Hospital, Harry Gwala Regional Hospital and Northdale Hospital (Pietermaritzburg hospital-complex) prior to the commencement of the study. Written informed consent was obtained from all individual participants involved in the study prior to enrolment (Appendix 1). Data collection booklets contained random reference numbers that were devoid of personal identifiers to maintain confidentiality of participant data.

## Results

All study participants were included in the data analyses. A total of 164 intern doctors were enrolled in the study, the majority of whom had graduated from medical school within the last 2 years. At the time of data collection, 63 (38.4%) were first-year interns rotating in internal medicine, paediatrics, obstetrics and gynaecology, or surgery, while 101 (61.6%) were second-year interns rotating in family medicine, anaesthesiology, psychiatry, or orthopaedics.

**FIGURE 1:**
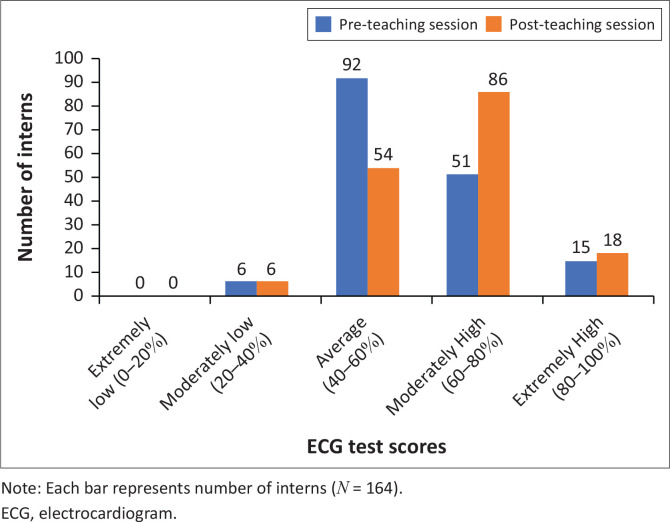
ECG test score percentage (%) ranges obtained pre-teaching and post-teaching.

**FIGURE 2:**
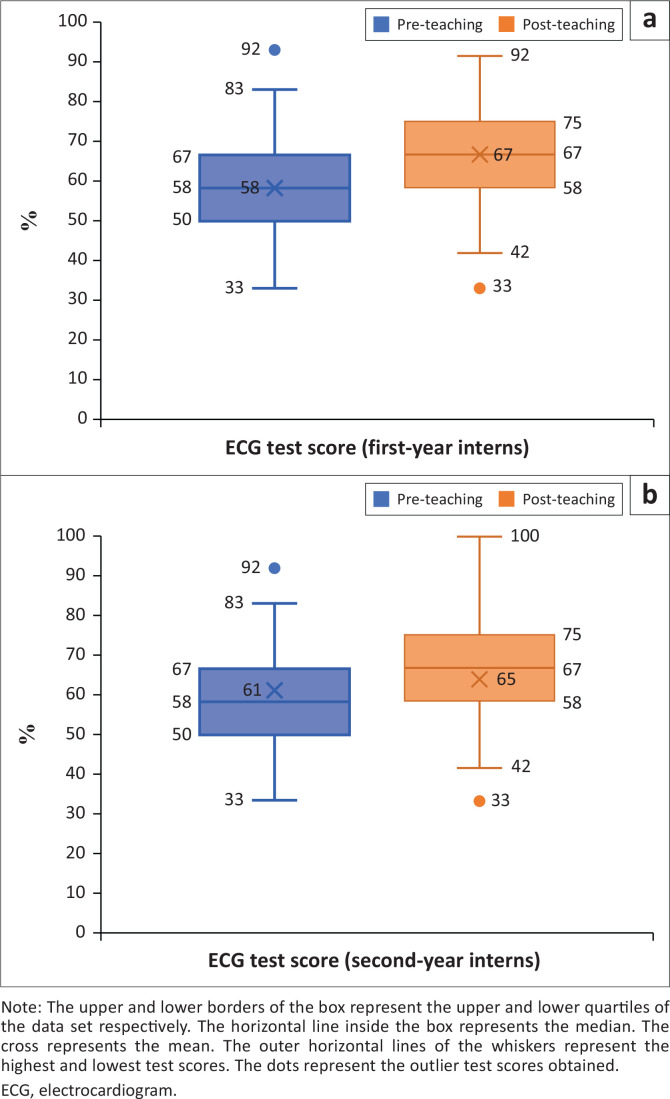
A box and whisker plot describing the distribution of ECG test scores of: (a) the first-year interns (*N* = 63); and (b) second-year interns (*N* = 101), obtained pre-teaching and post-teaching.

**FIGURE 3:**
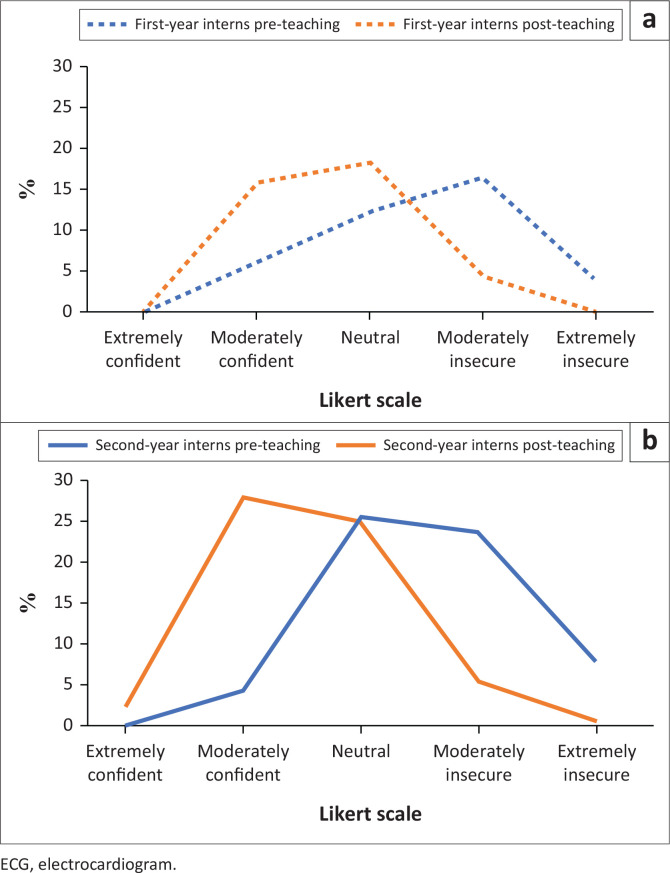
Self-confidence in electrocardiogram interpretation of: (a) first-year interns (*N* = 63); and (b) second-year interns (*N* = 101), as declared pre-teaching and post-teaching expressed in percentage (%).

In the pre-teaching ECG test, most interns scored between 40% and 60% (Figure 1), which in turn reflected baseline knowledge (overall mean test score 60.1%; overall median 58.3%). The highest individual test score obtained -pre-teaching was 91.7%, and the lowest score obtained was 33.3%. While the overall distribution of pre-teaching test scores was similar between the first-year interns and second-year interns, the mean test score was slightly higher for the second-year interns in comparison (Figure 2). In the pre-teaching self-confidence questionnaire, most interns (40.2%) declared themselves moderately insecure with ECG interpretation, followed by 37.8% who felt neutral, and no interns declared themselves extremely confident (Figure 3).

In the post-teaching ECG test, the majority scored within the 60% and 80% bracket (Figure 1). A Wilcoxon signed-rank test revealed that ECG interpretation test scores were overall higher after the teaching session (overall mean 65.8%, overall median 66.7%), and this was a statistically significant finding (*p* < 0.001). However, the effect size, as measured by partial eta squared, was small (ηp^2^ = 0.12), thereby indicating that the impact of the teaching session on the test scores was negligible. Individualised comparisons found that the teaching session made no difference to the test scores of 25 (15.3%) interns, and 45 (27.4%) interns performed worse after the teaching session. While the highest individual test score increased to 100% post-teaching, the lowest individual test score remained unchanged, and no interns scored in the extremely low category (0% – 20%) in either of the ECG tests (Figure 1). Interestingly, the first-year interns’ mean test score improved more in the post-teaching ECG test compared to the second-year interns (Figure 2). In the post-teaching self-confidence questionnaire, comparative analyses using a Wilcoxon signed-rank test revealed a statistically significant improvement on the Likert scale for 118 (72%) interns (*p* < 0.001); most interns (43.9%) declared themselves moderately confident, and 2.4% became extremely confident (Figure 3). However, 40 (24.4%) interns declared no difference in self-confidence, and six (3.7%) interns denoted a relative drop in self-confidence on the Likert scale after the teaching session.

Following the teaching session, the improved recognition of acute ischaemia on an ECG was only evidenced by certain ECG patterns (Table 1). There was a statistically significant improvement in the recognition of half of the ischaemic ECG patterns tested, with the most positive impact observed in the identification of inferior STEMI with posterior extension as an ECG pattern indicative of acute ischaemia (*p* < 0.001). However, there were still many interns who misinterpreted these four ischaemic ECG patterns as non-ischaemic post-teaching. In addition, albeit an insignificant finding, there was a mixed impact seen on the other four ischaemic ECG patterns, two of which resulted in decreased recognition, and no difference was made to the identification of Wellens Syndrome (Type A) as acute ischaemia post-teaching compared to pre-teaching. Conversely, the teaching session was also found to have a statistically significant positive impact on the recognition of two non-ischaemic ECG patterns. A normal sinus rhythm was erroneously identified as acute ischaemia by 23.2% before the teaching session, and this reduced to 14% post-teaching (*p* = 0.03). In addition, while the majority incorrectly identified BER as acute ischaemia in the pre-teaching tests (90.2%), this number decreased to 73.2% in the post-teaching tests (*p* < 0.001). However, acute pericarditis was more frequently misidentified as acute ischaemia by interns after the teaching session compared to before (*p* < 0.001). Overall, this study’s teaching session was observed to improve the interns’ ability to correctly identify true acute ischaemia on an ECG more compared to correctly identifying a non-ischaemic ECG. Sensitivity improved from 66% pre-teaching to 74% post-teaching, and specificity improved from 47% pre-teaching to 49% post-teaching. Sensitivity was calculated by dividing the total number of true ischaemic ECGs correctly identified by the number of true ischaemic ECGs correctly identified added to the number of true ischaemic ECGs incorrectly identified as non-ischaemic. Specificity was calculated by dividing the total number of non-ischaemic ECGs correctly identified by the number of non-ischaemic ECGs correctly identified added to the number of non-ischaemic ECGs incorrectly identified as ischaemic.

**TABLE 1:** The impact of the teaching session on the interns’ ECG test answers categorized by ECG diagnosis.

ECG diagnosis	Test answer submitted	Pre-teaching*	Post-teaching*	*p*-value**
Normal Sinus Rhythm	-	-	-	0.028
	Ischaemia	38	23	-
	Non-ischaemic (CORRECT ANSWER)	126	141	-
Benign early repolarization	-	-	-	< 0.001
	Ischaemia	148	120	-
	Non-ischaemic (CORRECT ANSWER)	16	44	-
Ventricular-paced rhythm (Modified Sgarbossa-Smith Negative)	-	-	-	0.902
	Ischaemia	106	104	-
	Non-ischaemic (CORRECT ANSWER)	58	60	-
Acute pericarditis	-	-	-	< 0.001
	Ischaemia	55	89	-
	Non-ischaemic (CORRECT ANSWER)	109	75	-
Inferior STEMI with right ventricular involvement	-	-	-	0.002
	Ischaemia (CORRECT ANSWER)	139	155	-
	Non-ischaemic	25	9	-
Wellens Syndrome (Type A)	-	-	-	1.000
	Ischaemia (CORRECT ANSWER)	117	117	-
	Non-ischaemic	47	47	-
NSTEMI involving antero-lateral and inferior territories	-	-	-	0.885
	Ischaemia (CORRECT ANSWER)	47	45	-
	Non-ischaemic	117	119	-
Lateral STEMI	-	-	-	0.473
	Ischaemia (CORRECT ANSWER)	143	138	-
	Non-ischaemic	21	26	-
Hyperacute T-waves	-	-	-	0.755
	Ischaemia (CORRECT ANSWER)	136	139	-
	Non-ischaemic	28	25	-
Wellens Syndrome (Type B)	-	-	-	0.011
	Ischaemia (CORRECT ANSWER)	79	100	-
	Non-ischaemic	85	64	-
LBBB (Modified Sgarbossa-Smith Positive)	-	-	-	< 0.001
	Ischaemia (CORRECT ANSWER)	113	142	-
	Non-ischaemic	51	22	-
Inferior STEMI with posterior extension	-	-	-	< 0.001
	Ischaemia (CORRECT ANSWER)	98	132	-
	Non-ischaemic	66	32	-

STEMI, ST-elevation myocardial infarction; NSTEMI, non-ST-elevation myocardial infarction; LBBB, left bundle branch block; ECG, electrocardiogram.

* , number of interns who submitted the answer specified (*N* = 164);

** , represents the difference made by the teaching session on the test answer submitted post-teaching compared to pre-teaching; calculated using McNemar Chi-square test applying binomial distribution; *p*-value ≤ 0.05 considered significant.

Overall, 90.9% of interns indicated that the teaching session was helpful, and only 1.8% found it immensely unhelpful. Lastly, the majority of interns (99.4%) expressed a desire to receive more formal teaching sessions around ECG topics in general during their internship training.

## Discussion

This group of interns scored just above-average in identifying acute myocardial ischaemia on an ECG at baseline, thereby aligning with our hypothesised gap in skill proficiency among South African intern doctors. Interestingly, this level of performance compares similarly to interns and junior doctors training in other countries. A recent study conducted in Saudi Arabia reported an ECG interpretation mean accuracy score of 54% among their intern doctors; however, this study was much larger (*N* = 373); it tested a wider range of ECG diagnoses besides acute myocardial ischaemia, and it used a different methodology to quantify competency compared to our study.^8^ Another study conducted in Iran (*N* = 323) reported an overall ECG competency score of 51.3% with 63.8% of its participants demonstrating an inability to accurately identify acute myocardial infarction.^16^ Another study conducted in the United Kingdom (*N* = 62) revealed an even lower mean proficiency score of 45% among its doctors.^7^ However, unlike our study, these studies from Iran and the United Kingdom did not restrict their assessment to intern doctors exclusively. Another study that solely evaluated newly graduated doctors awaiting internship placement at the University of Nairobi (*N* = 177) revealed an overall ECG competency score of 19.2%.^10^ Surprisingly, more than 66% of participants in this Kenyan study declared themselves ‘somewhat confident’ in their skills, and only 21.6% requested additional ECG training. This was unlike our study, where most of our interns declared themselves moderately insecure with their skills at baseline, and the majority requested additional ECG teaching during internship training. In reference to the limited research available, we were able to extrapolate that ECG interpretation proficiency gaps exist commonly among interns and junior doctors worldwide. We therefore identified an opportunity for skill enhancement using a standardised ECG teaching session.

Electrocardiogram interpretation education strategies have been extensively studied, and there is an ongoing debate on how to teach this skill more effectively. A systematic review and meta-analysis of 59 studies among physicians found that computer-assisted instruction was favoured over face-to-face instruction, group training was favoured over individual training, and peer instruction was favoured over faculty-led instruction.^5^ The value of computer-assisted instruction was further explored among both medical students and specialist trainees, and these studies suggested that computer-assisted instruction is not specifically superior to other approaches, but it did positively impact ECG knowledge acquisition when used as a supplement to face-to-face instruction.^17^ A ‘flipped classroom’ pedagogical approach to ECG education was also studied previously, and findings appeared positive among medical students.^18^ This is an approach where the learner engages in self-study of the academic content provided by the teacher prior to attending a teacher-led face-to-face discussion.^18^ While some studies suggest that ECG workshops and courses that apply a ‘flipped classroom’ model are favoured by medical students and emergency medicine residents,^19,20^ other studies found that participation in extracurricular ECG courses did not necessarily correlate with better competency scores among intern doctors.^8,11^ Electrocardiogram teaching strategies were also explored by local studies among undergraduate medical students. A qualitative study conducted at the University of the Free State (Free State province, South Africa) revealed that students preferred small-group tutorials that integrated computer-based animations for ECG interpretation education.^21^ Another study conducted at the University of Cape Town (Western Cape province, South Africa) demonstrated that combining conventional lectures with web-based computer-assisted instruction that students could view in advance improved baseline competency and self-confidence more compared to attending the lectures alone.^22^ The ECG teaching session used in our study was computer-assisted and lecture-based as this educational approach was practical to execute and easily reproducible. It incorporated some positive elements mentioned in previous research; however, its poor sensitivity and specificity performance echo its inadequacies, and our analyses yielded mixed results.

While the mean ECG test scores did increase following our ECG teaching session, the overall difference was marginal, and the majority of interns demonstrated improvement by identifying only one additional ECG accurately for acute ischaemia. Errors in accuracy persisted with interns still missing true ischaemia on some ECGs and misinterpreting other non-ischaemic ECG traces as acute ischaemia, including a normal sinus rhythm. Acute pericarditis was the most frequently misidentified as acute ischaemia post-teaching compared to pre-teaching. However, it is noteworthy that our teaching session could have introduced cognitive bias to a certain degree by including a stepwise approach on how to rule in acute ischaemia on an ECG with ST-elevation before searching for ECG changes suggestive of acute pericarditis.^23^ These results suggest a critical need for more robust ECG training during internship, emphasising not only the recognition of acute ischaemia, but also a revision of basic ECG interpretation skills. Given that our teaching session was found to have a more positive impact on the first-year interns compared to the second-year interns, this additional analysis provides further insight into whether targeted teaching interventions should be directed at undergraduate medical training for first-year interns or local facility teaching practices for second-year interns.

In comparison, the impact of our ECG teaching session on the interns’ baseline self-confidence levels was mostly positive. This dyssynchronous impact on ECG test scores compared to self-confidence levels may reflect a significant challenge associated with ECG education. While there is no gold standard approach for teaching ECG interpretation, enhancing our teaching strategy with a ‘flipped classroom’ component may have resulted in better post-teaching test scores. However, more research is needed to define an effective strategy that teaches ECG interpretation skills without triggering overestimation of competency levels or exacerbating apprehension among intern doctors. Furthermore, given the complexity of this skill and the broad scope of life-threatening diagnoses that rely on accurate ECG interpretation for timeous identification, our results suggest that regular competency assessments are justified so that critical knowledge gaps are addressed prior to the completion of internship training.

To our knowledge, this was the first study that assessed ECG interpretation accuracy and associated self-confidence in skill levels among intern doctors training in South Africa, thereby adding a local perspective to a widely researched topic. However, there are some limitations to acknowledge when considering the results. Given that this study was confined to intern doctors specifically rotating within the Pietermaritzburg hospital-complex, the pre-teaching results may not reflect the baseline ECG interpretation accuracy and self-confidence levels of all South African intern doctors. This study’s ECG interpretation tests also just assessed identification of acute myocardial ischaemia as a binary parameter to allow for more efficient data collection, whereas this skill is known to be much more complex. A more reliable method to measure an intern’s ability to identify acute myocardial ischaemia on an ECG using a systematic approach may be to evaluate their analytical reasoning open-endedly. A future study that incorporates qualitative or tiered scoring assessment metrics may uncover a more meaningful evaluation of ECG interpretation competency that extends beyond rapid visual pattern recognition. In addition, the pre-teaching and post-teaching ECG tests were all conducted on the same day as the teaching session. Therefore, the results obtained in the post-teaching assessments may not necessarily reflect this group of interns’ knowledge and self-confidence levels in the long term. A delayed follow-up study will hence be valuable to evaluate retention of ECG interpretation knowledge gained from the teaching session. Furthermore, with this study falling short of its target sample size, the effect of the teaching session on the interns’ baseline ECG interpretation accuracy and self-confidence levels should be interpreted with caution.

## Conclusion

This sample of intern doctors mostly demonstrated above-average accuracy in identifying acute myocardial ischaemia on an ECG, and the majority declared themselves moderately insecure with their skill level at baseline. While a standardised teaching session improved self-confidence overall from moderately insecure to moderately confident, errors in interpretation accuracy persisted and, in some cases, worsened. These mixed results not only echo the challenge of acquiring this complex skill as a junior doctor but also the heterogeneous impact of standardised teaching strategies. Self-awareness of skill level should ideally drive an intern’s knowledge-seeking behaviour, but mismatched self-confidence could go unnoticed without appropriate supervision. Our study suggests that ongoing ECG training during internship is important and that regular competency assessments are justified to close critical knowledge gaps prior to the completion of internship training in South Africa.

## Appendix 1: Combined participant informed consent and non-disclosure agreement.

Study participant informed consent

Dear Colleague,

My name Dr Relisha K. Naidoo, an Emergency Medicine Registrar from the University of KwaZulu-Natal, Department of Health Sciences, Division of Emergency Medicine, Mobile +27 76 138 7973, Email: relisha.naidoo@gmail.com. You are being invited to consider participating in a study that involves research entitled:

***Recognition of acute myocardial ischaemia on an electrocardiogram by junior doctors; can formal training improve performance? A prospective descriptive analytical study at a state hospital-complex in KwaZulu-Natal.***

The aim of this research is to assess whether the recognition of acute myocardial ischaemia on an electrocardiogram (ECG) by junior doctors can be improved by a recap training session. The study is expected to enrol a total of 200 intern doctors rotating within the Pietermaritzburg Hospital complex.

It will involve a pre-training and post-training questionnaire and ECG interpretation assessment. The recap training session will revise with you how to recognise acute myocardial ischaemia on an ECG using a systematic approach. The duration of your participation if you choose to enrol and remain in the study is expected to be 1 h. This study will honour participant anonymity throughout its data collection process. We hope that the study will raise awareness on your competency and self-confidence levels with ECG interpretation and identification of acute myocardial ischaemia/infarction in an adult. Should the recap training session be found useful to achieve this goal, we hope that this study will encourage further similar training to be offered during internship for future junior doctors.

To the knowledge of the researcher, this research does not expose the participant to any foreseeable risk. This study has been ethically reviewed and approved by the UKZN Biomedical Research Ethics Committee (approval number BREC/00005193/2023).

In the event of any problems or concerns/questions, you may contact the researcher at Mobile +27 76 138 7973, Email: relisha.naidoo@gmail.com; or the UKZN Biomedical Research Ethics Committee (see contact details at the end of this document).

Participation in this research is strictly voluntary, and participants may withdraw participation at any point, and that in the event of refusal/withdrawal of participation, the participants will not incur penalty or other benefit to which they are normally entitled.

Should the participant request for withdrawal from the study on the day of their data collection, the participant’s contribution will be discarded. Should the participant request for withdrawal from the study after the day of their data collection, please note that it will be nearly impossible to discard the participant’s contribution due to the anonymity of the participant during the data collection process.

No costs will be incurred by participants because of participation in this study. There are no incentives or reimbursements for participation in this study.

To protect confidentiality of personal and clinical information, all data collection documentation will not include participant identification.

### Study participant non-disclosure agreement

#### Parties

This Non-Disclosure Agreement (hereinafter referred to as the ‘Agreement’) is entered into on ____________________ (the ‘Effective Date’), by and between Dr Relisha K. Naidoo, with HPCSA Registration MP0782548, (hereinafter referred to as the ‘Disclosing Party’) and________________________________, with HPCSA Registration ________________________, (hereinafter referred to as the ‘Receiving Party’) (collectively referred to as the ‘Parties’).

### Confidential information

The Receiving Party agrees not to disclose, copy, clone, or modify any confidential information related to the Disclosing Party and agrees not to use any such information without obtaining consent.

‘Confidential information’ refers to any data and/or information that is related to theDisclosing Party, in any form, including, but not limited to, oral or written. Such confidential information includes, but is not limited to, any information related to the business or industry of the Disclosing Party, such as discoveries, processes, techniques, programs, knowledge bases, customer lists, potential customers, business partners, affiliated partners, leads, know-how, or any other services related to the Disclosing Party.

### Return of confidential information

The Receiving Party agrees to return all the confidential information to the Disclosing Party upon the termination of this Agreement.

### Ownership

This Agreement is not transferable and may only be transferred by written consent provided by both Parties.

### Governing law

This Agreement shall be governed by and construed in accordance with the laws of the University of KwaZulu-Natal.

--------------------------------------------------------------------------------------------------

I ______________________________________________ have been informed about the study entitled ‘***Recognition of acute myocardial ischaemia on an electrocardiogram by junior doctors; can formal training improve performance? A prospective descriptive analytical study at a state hospital-complex in KwaZulu-Natal’*** by Dr Relisha K. Naidoo.

I understand the purpose and procedures of the study. I have been given an opportunity to answer questions about the study and have had answers to my satisfaction. I declare that my participation in this study is entirely voluntary and that I may withdraw at any time without affecting any treatment or care that I would usually be entitled to.

If I have any further questions/concerns or queries related to the study, I understand that I may contact the researcher at Mobile +27 76 138 7973, Email: relisha.naidoo@gmail.com. If I have any questions or concerns about my rights as a study participant, or if I am concerned about an aspect of the study or the researchers, then I may contact:

### Biomedical research ethics administration

Research Office, Westville Campus

Govan Mbeki Building

Private Bag X 54001

Durban

4000

KwaZulu-Natal, South Africa

Tel: 27 31 2604769 - Fax: 27 31 2604609

Email: BREC@ukzn.ac.za

The Parties hereby agree to the terms and conditions set forth in this Informed Consent and Non-disclosure Agreement and such is demonstrated by their signatures below:

### Disclosing party

**Name:** Dr Relisha K. Naidoo

Signature: ________________ Date: _________

### Receiving party

____________________   -------------------

__________

Name of Participant   Signature of Participant  Date

____________________   _____________________

Signature of Witness   Date

(If applicable)

____________________  _____________________

Signature of Translator  Date

(If applicable)
